# The Nutritional Quality of Kids’ Menus from Cafés and Restaurants: An Australian Cross-Sectional Study

**DOI:** 10.3390/nu14132741

**Published:** 2022-06-30

**Authors:** Gina S. A. Trapp, Claire E. Pulker, Miriam Hurworth, Kristy K. Law, Sally Brinkman, Christina M. Pollard, Amelia J. Harray, Ros Sambell, Joelie Mandzufas, Stephanie Anzman-Frasca, Siobhan Hickling

**Affiliations:** 1 Telethon Kids Institute, The University of Western Australia, P.O. Box 855, West Perth, Perth, WA 6872, Australia; miriam.hurworth@telethonkids.org.au (M.H.); sally.brinkman@telethonkids.org.au (S.B.); amelia.harray@telethonkids.org.au (A.J.H.); joelie.mandzufas@telethonkids.org.au (J.M.); 2 East Metropolitan Health Service, Kirkman House, 20 Murray Street, East Perth, Perth, WA 6004, Australia; claire.pulker@health.wa.gov.au (C.E.P.); kristy.law@health.wa.gov.au (K.K.L.); 3 School of Population Health, Curtin University, Kent Street, GPO Box U1987, Perth, WA 6845, Australia; c.pollard@curtin.edu.au; 4 Institute of Nutrition Research, School of Medical and Health Sciences, Edith Cowan University, 270 Joondalup Drive, Joondalup, WA 6027, Australia; r.sambell@ecu.edu.au; 5 School of Population and Global Health, The University of Western Australia, 35 Stirling Highway, Perth, WA 6009, Australia; siobhan.hickling@uwa.edu.au; 6 Department of Pediatrics, Jacobs School of Medicine and Biomedical Sciences, University at Buffalo, 12 Capen Hall, Buffalo, NY 14260-1660, USA; safrasca@buffalo.edu

**Keywords:** Kids’ Menus, cafés, restaurants, children, nutritional quality

## Abstract

Australian families increasingly rely on eating foods from outside the home, which increases intake of energy-dense nutrient-poor foods. ‘Kids’ Menus’ are designed to appeal to families and typically lack healthy options. However, the nutritional quality of Kids’ Menus from cafes and full-service restaurants (as opposed to fast-food outlets) has not been investigated in Australia. The aim of this study was to evaluate the nutritional quality of Kids’ Menus in restaurants and cafés in metropolitan Perth, Western Australia. All 787 cafes and restaurants located within the East Metropolitan Health Service area were contacted and 33% had a separate Kids’ Menu. The validated *Kids’ Menu Healthy Score (KIMEHS)* was used to assess the nutritional quality of the Kids’ Menus. Almost all Kids’ Menus (99%) were rated ‘unhealthy’ using KIMEHS. The mean KIMEHS score for all restaurants and cafés was −8.5 (range −14.5 to +3.5) which was lower (i.e., more unhealthy) than the mean KIMEHS score for the top 10 most frequented chain fast-food outlets (mean −3.5, range −6.5 to +3). The findings highlight the need for additional supports to make improvements in the nutritional quality of Kids’ Menus. Local Government Public Health Plans provide an opportunity for policy interventions, using locally relevant tools to guide decision making.

## 1. Introduction

Poor diet quality and weight gain have become normalized in Australia, with the latest national nutrition survey reporting that energy-dense, nutrient-poor foods, contribute 41% and 35% of daily energy intake for children and adults, respectively [1]. In 2017–2018, one-quarter of Australian children, and two-thirds of Australian adults were classified as overweight or obese [1]. Being overweight or obese in childhood often continues into adulthood, which is associated with both early mortality and co-morbidities later in life [2]. Therefore, addressing excess weight gain during childhood is a public health priority [3].

Food environments influence food choice via the type, location and accessibility of food outlets in a local neighborhood and within-store marketing including product, price, promotion, placement, and provision of information [4]. Obesogenic food environments are common in Australia and promote the intake of energy-dense nutrient-poor foods, which reduces diet quality and contributes to increases in excess energy intake, weight gain, obesity and diet-related chronic diseases [5]. Food preferences and eating behaviours are established early in life [3] and studies indicate eating away from home is associated with childhood obesity [6]. Food environments that support healthy choices are needed to improve children’s diet quality. This is highlighted in the newly released National Obesity Strategy (Australia) [7], which focuses on increasing access to healthy food and drinks. This Strategy also highlights the dangers in heavily promoted unhealthy foods and drinks within communities, which can undermine public health efforts to choose healthy foods.

Increasingly, Australian families rely on purchasing food prepared outside of the home, with restaurant tables turning into the “new generation” of the dinner table where families connect [8]. Eating meals away from home and from fast-food outlets account for a third of all food expenditure [9]. In the period 2015–2016, Australian households spent on average AUD 44 per week in restaurants, an increase of AUD 12 per week from 2009–2010, and an additional AUD 31 on takeaway meals [10].

Food establishments offering a “Kids’ Menu” appeal to families as it suggests a child-friendly environment [11,12]. Kids’ Menus (or Children’s Menus) are generally designed for children under 12 years and typically lack nutritious choices such as salads, whole-grain products, and fruit-based desserts [13]. Instead, Kids’ Menus include foods high in fat and salt, like chicken nuggets and French fries, with sugar-sweetened beverages often bundled in as the default drink option [14,15]. Portion sizes are often larger than would be recommended for children [16,17] and can encourage overconsumption of energy [18,19,20]. Children who frequently eat out have been shown to have higher intakes of energy, fat, sodium, and added sugar than their peers [21]. 

### 1.1. Nutritional Quality of Food Service Menus in Australia

To date, few Australian studies have investigated the impact of food-service environments on children’s eating behaviours [22]. Assessment of the nutritional quality of major fast-food chains’ Kids’ Menus in Australia found substantial improvements were needed [23]. For example, the nutrient content of 199 kids’ meal combinations from six major fast-food chains in New South Wales exceeded 30% of the estimated daily requirements for energy, saturated fat, added sugar, and sodium in a single meal [24]. The nutritional content of Kids’ Menus from full-service, sit down, restaurants and cafés have not been explored in Australia. Thus, it is currently unknown whether the meals promoted to children in cafés and restaurants differ in nutritional quality when compared with fast-food chains. 

### 1.2. Evaluating Neighbourhood Food Environments to Inform Local Policy Response

Addressing the environmental drivers of overweight and obesity is a key focus of the East Metropolitan Health Service’s (EMHS) Obesity Prevention Strategy (the Strategy) in Perth, Western Australia (WA). The EMHS aims to maintain and improve the health of 725,500 people residing within the geographic area [25]. The Strategy adopts a public health approach and includes 28 co-delivered reinforcing actions. To ensure action is tailored to the EMHS context, there is a commitment to building a portfolio of local and up-to-date intelligence to continuously inform policy action. This includes evaluating the dietary risk of neighborhood food outlets [26]. 

The aim of this study was to evaluate the nutritional quality of Kids’ Menus in full-service restaurants and cafés located within the EMHS geographic area in Perth, WA. The two research questions were: (1) What is the nutritional quality of Kids’ Menus in cafés and full-service restaurants in the EMHS geographic area? and (2) Does the nutritional quality of Kids’ Menus differ between cafés and restaurants, and fast-food outlets?

## 2. Materials and Methods

### 2.1. Sample Selection

A list of all food businesses located within the EMHS area were obtained from 13 Local Governments (LGs). These lists were used to identify and include every café (defined as establishments selling food and beverages to customers on the premises during the day only) and full-service restaurant (defined as establishments selling food and beverages to customers on the premises, where dinner is served and there is table service) into the study.

### 2.2. Obtaining Kids’ Menus from Cafés and Restaurants

All 787 cafés and restaurants located within the EMHS area were contacted in April/May 2021, either by phone or email to obtain the Kids’ Menu, defined as a menu or section of the menu that specifically targets diners under the age of 18 years [13], using terms such as Kids’ Menu, Children’s Menu, Kids’ Corner, Especially for Kids. For cafés, the lunchtime Kids’ Menu was obtained, and for restaurants the evening Kids’ Menu was obtained. Cafés and restaurants without a separate Kids’ Menu were recorded. The main menu for all cafés and restaurants was also obtained to determine whether children had the opportunity to order any main menu items at a reduced price and/or portion. 

### 2.3. Kids’ Menus from the Top 10 Fast-Food Chains

A fast-food outlet was defined as a food business where food is ordered at the counter, can be eaten without cutlery, and served immediately [27]. Kids’ Menus from the top ten most frequented fast-food chain outlets in Australia, as identified by Roy Morgan industry data [28], were included: McDonald’s™ (14 May 2021), KFC™ (14 May 2021), Subway™ (14 May 2021), Hungry Jack’s™ (14 May 2021), Domino’s Pizza™ (14 May 2021), Red Rooster™ (14 May 2021), Grill’d™ (14 May 2021), Nando’s™ (14 May 2021), Pizza Hut™ (14 May 2021), and Noodle Box™ (14 May 2021). Kids’ Menus were downloaded from the business’ website. 

### 2.4. Kids’ Menu Assessment

The following information was recorded from each Kids’ Menu: total number and price of main meals, side dishes, beverages and desserts; description of each main meal, side dish, beverage and dessert offered; main meal healthy default side option (i.e., foods aligned with the Australian Dietary Guidelines [29], such as salad, vegetables, wholegrains), substitution for a healthier default side allowed (yes/no); milk and/or water offered (yes/no); presence of ‘meal deals’ (i.e., main meals/desserts/drinks bundled together), and, if yes, whether healthy/unhealthy options are included by default; marketing strategies present (cartoon characters, toys, logos, brand names, other); nutritional information present (energy/fat/sodium/fibre content, allergens, healthier cooking methods), symbol or words denoting healthful options (‘healthy choice’, ‘low fat’, ‘lean’, ‘light’, ‘low-calorie’).

#### 2.4.1. Nutritional Assessment via the Traffic Light System

The Traffic Light System of food classification (i.e., green, amber, red), based on the Healthy Options WA Food and Nutrition Policy [30], was used to assess each Kids’ Menu item according to its nutrient content, and alignment with the recommendations of the Australian Guide to Healthy Eating [29]. Foods classified as Green are from one or more of the five food groups (i.e., fruit, vegetables, dairy and alternatives, meat and alternatives, and grain or cereal foods) recommended for everyday consumption and are good sources of nutrients [31]. The Australian Dietary Guidelines state that intake of foods containing saturated fat, added salt, added sugars and alcohol should be limited [29]. Therefore, foods classified as Amber have some nutritional value, but may contain moderate amounts of energy, fat, sugar and/or salt, and should be selected carefully [31]. Foods classified as Red are not an essential part of a healthy diet, are often high in energy, fat, sugar and/or salt, and represent discretionary items in the Australian Guide to Healthy Eating [29,32]. All main meals, sides, beverages, and desserts listed on Kids’ Menus were classified as either Red, Amber, or Green, by an EMHS dietitian with extensive experience in applying the Traffic Light System.

#### 2.4.2. Nutritional Assessment via the Kids’ Menu Healthy Score (KIMEHS)

KIMEHS is a validated tool designed to assess the nutritional quality of Kids’ Menus obtained from restaurant websites, in-store display menus, or table menus [33]. Based on adherence to the Mediterranean dietary pattern, which is widely recognised as being healthy due to the high intake of plant-based foods [34,35], the KIMEHS assesses the presence of major food groups, including vegetables, pulses, cereals, meat, fish and eggs, fruit, and water. The KIMEHS excludes dairy products and fats due to difficulty in retrieving adequate information from the menu description. 

Each Kids’ Menu was evaluated using the KIMEHS tool [33], which applies positive points for healthy options and negative scores for non-healthy options. The magnitude of the attributed points is proportional to the impact that the food option has on menu quality and health [33]. A total KIMEHS score is calculated across the whole menu, with a possible range from −17 to 17. Total KIMEHS scores can be further divided into 5 categories ranging from ‘unhealthy’ (−17 to 0.49) to ‘moderately unhealthy’ (0.50 to 5.49) to ‘going healthy’ (5.50 to 11.49) to ‘moderately healthy’ (11.50 to 13.49) to ‘healthy’ (13.50 to 17).

### 2.5. Statistical Analysis

Data were analysed using the SPSS for Windows statistical software package version 24 (IBM Corp. Released 2016. Armonk, NY, USA: IBM Corp USA). The frequency of availability of kids’ menus was compared between cafes and restaurants. The number and price of main meals, side dishes, beverages, and desserts available on Kids’ Menus was compared between cafes and restaurants. Applying the Healthy Options WA Policy traffic light nutrient criteria, the proportion of Green, Amber, and Red, classified Kids’ Menu items were summarised by food business type. The Kids’ Menu Healthy Score (KIMEHS) score was calculated and categorized by food business type. The traffic light rating and KIMEHS score were also calculated for each of the top 10 fast-food chains.

## 3. Results

### 3.1. Proportion of Cafés and Restaurants with a Kids’ Menu

Table 1 presents the proportion of cafés and restaurants with a separate Kids’ Menu. Of the 787 food businesses identified, 33% had a separate Kids’ Menu, the proportion was higher for cafés than restaurants (38% vs. 28%, respectively). The proportion of food businesses without a separate Kids’ Menu where children could order anything from the main menu at a reduced price and/or portion, was 14% (cafés 16%, restaurants 12%).

### 3.2. Main Meals Offered on Kids’ Menus

Kids’ Menus offered an average of 4.6 main meals, at an average price of AUD 10.15 (range AUD 3–28) (Table 2). The average price of main meals was higher in restaurants compared with cafés (AUD 12.16 vs. AUD 9.10, respectively). Restaurant Kids’ Menus had a higher proportion of main meals with a healthy default side (i.e., salad or vegetables) compared to café Kids’ Menus (18% vs. 2%, respectively). Five food businesses offered to substitute an unhealthy default side for a healthy default side on their menu, and one charged a higher price for the swap.

The top five most frequent main meals present on Kids’ Menus from cafés were: fried chicken nuggets and chips (present on 68% of menus), egg on toast variations (49%), toasted sandwiches (45%), fish and chips (44%) and pasta dishes (22%). The top five most frequent main meals present on Kids’ Menus from restaurants were: pasta dishes (54%), fried chicken nuggets and chips (53%), fish and chips (47%), burger and chips (21%), pizza (18%).

### 3.3. Additional Side Dishes Offered on Kids’ Menus

On average, 9% of food businesses offered an additional side dish for purchase, with an average price of AUD 6.35 per side dish (range AUD 3.90 to AUD 10.50) (Table 2). The proportion of Kids’ Menus offering additional side dishes was higher for restaurants than cafés (13% vs. 5%, respectively). Additional side dish options on Kids’ Menus were (in order of frequency), chips or wedges, kid’s garden salad and a selection of vegetable crudites.

### 3.4. Beverages Offered on Kids’ Menus

Around half (54%) of Kids’ Menus offered one beverage type or more. The average beverage price was AUD 3.70 (range AUD 1.00 to AUD 7.50) and was slightly higher for restaurants than cafés (AUD 4.20 vs. AUD 3.70) (Table 2). Water was offered on 1.5% of Kids’ Menus and plain milk offered on 7% of Kids’ Menus (12% in cafes 1%, in restaurants). No food businesses offered free refills on sugar sweetened beverages. The top 10 most prevalent beverages on Kids’ Menus (in order of frequency) were: juices, milkshakes, babycinos (i.e., typically a drink of frothy milk with chocolate topping designed as an alternative to coffee for young children), hot chocolates, soft drinks, fresh plain milk, fruit smoothies, water, and spiders (i.e., a chilled drink consisting of ice cream in soft-drink or a mixture of flavored syrup and carbonated water).

### 3.5. Desserts Offered on Kids’ Menus

Over half (58%) of all Kids’ Menus offered one dessert or more. The average price of a dessert on a Kids’ Menu was AUD 6.08 (range AUD 1.50 to AUD 14.00) and was similar for restaurants and cafés (Table 1). The top 10 most prevalent desserts on Kids’ Menus (in order of frequency) were: pancakes, ice cream/sundaes, fairy bread (i.e., bread with margarine or butter topped with colorful sugar sprinkles), waffles, fruit, biscuits/cookies, chocolate cake/brownies, chocolate freckle lollipops, churros, and other.

### 3.6. Promotional ‘Meal Deals’ on Kids’ Menus

More than a third (35%) of all Kids’ Menus offered a meal deal (Table 2), with the proportion higher for restaurants than cafés (37% vs. 33%, respectively). The most common type of meal deal offered was the inclusion of a drink with the main meal. Kids’ Menus from restaurants had a higher proportion of meal deals that included an unhealthy beverage (26% vs. 10%) or dessert (20% vs. 15%) by default than cafes, respectively. No meal deals specified that a healthier dessert could be substituted, and only 1.5% specified a healthy beverage could be substituted.

### 3.7. Marketing Strategies Used on Kids’ Menus

Around one in five Kids’ Menus (19%) featured a logo or brand name (Table 2). A toy or ‘surprise’ was offered on 9% of Kids’ Menus and 19% of Kids’ Menus featured ‘other’ marketing strategies, such as the use of bright colors and pictures, or a coloring-in pack gift.

### 3.8. Nutrition Information Provided on Kids’ Menus

One in four Kids’ Menus (23%) included descriptions of healthier cooking methods and 20% included allergen information (Table 2). One per cent of Kids’ Menus identified healthy menu items using a symbol or word. Thirteen per cent of Kids’ Menus displayed the energy (kilojoule) content of items (mostly chain food businesses) and 2% displayed other nutritional information (e.g., fat, sodium, fiber). Cafés had a higher proportion of Kids’ Menus displaying energy content (19% vs. 4%) and healthier cooking methods (33% vs. 11%), compared with restaurants.

### 3.9. Nutrition Assessment via the Traffic Light System

Table 3 presents the proportion of Green, Amber, and Red, classified Kids’ Menu items by food business type. Most (70%) Kids’ Menu items were classified as Red; with 73% of main meals, 90% of separate side dishes, 58% of beverages and 70% of desserts classified as Red. For main meals, restaurants offered a higher proportion of Green main meals (31% vs. 22%) and a lower proportion of Red main meals (66% vs. 77%), compared with cafés. Cafés had a higher proportion of beverages classified as Green, compared with restaurants (29% vs. 8%). Restaurants had a higher proportion of desserts classified as Red compared with Cafés (94% vs. 65%). 

### 3.10. Nutrition Assessment via the Kids’ Menu Healthy Score (KIMEHS)

Table 4 presents total KIMEHS scores and categories by business type. Almost all (99%) food businesses had a KIMEHS score in the Unhealthy category (mean score −8.5, range −14.5 to 3.5). Only two restaurants had a KIMEHS score in the Moderately Unhealthy category and no food businesses had a KIMEHS score in the Going Healthy, Moderately Healthy or Healthy categories. 

### 3.11. Nutrition Assessment of Kids’ Menus from the Top 10 Fast-Food Chains Compared with Cafés and Restaurants

Kids’ Menus from the top 10 fast-food chains had higher proportions of Green, and lower proportions of Red, classified main meals, separate sides, and beverages (as well as all Kids’ Menu items combined) than Kids’ Menus from cafés and restaurants (Table 5). 

The average KIMEHS score for the top 10 fast-food chains was −3.5 (range −6.5 to 3) which is higher (i.e., less unhealthy) than the average KIMEHS score for all cafés and restaurants in the EMHS combined (−8.5, range −14.5 to 3.5) (Table 6). 

## 4. Discussion

This study appears to be the first in Australia to describe and evaluate the nutritional quality of Kids’ Menus from full service, sit-down restaurants, and cafés. We found, on average, one-third of cafés and restaurants had a separate Kids’ Menu. Overall, Kids’ Menus lacked variety, rarely offered main meals with a healthy default side (i.e., salad/vegetables), rarely offered water or plain milk as a beverage option and presented little, if any, nutritional information. Furthermore, Kids’ Menus did not identify or promote more healthful options or use descriptions of healthier cooking methods. The most frequent meals offered were generally all discretionary foods, such as chicken nuggets and chips, burger and chips, and fish and chips, with sugar-sweetened beverages often the default drink. When applying the Traffic Light System of food classification, the vast majority (70%) of all Kids’ Menu items were classified as Red, denoting they were discretionary foods. When applying the KIMEHS scoring tool, 99.2% of all cafés and restaurants scored in the ‘Unhealthy’ category. Whilst there were some differences in nutritional quality between cafés and restaurants (i.e., Kids’ Menus from restaurants scored slightly better in terms of nutritional quality than Kids’ Menus from cafés), overall, our findings highlight the nutritional quality of Kids’ Menus in both cafes and restaurants is very poor. 

This study also found the nutritional quality of Kids’ Menus from the top ten chain brand fast-food outlets was slightly better than Kids’ Menus from cafés/restaurants. Kids’ Menus from the top 10 fast-food chains had higher proportions of healthier Green and lower proportions of unhealthy Red main meals, separate sides, and beverages than those from cafés and restaurants. The average KIMEHS score for the top 10 fast-food chains was higher (i.e., healthier) than the average for all cafés and restaurants. Overall, our findings suggest that whilst Kids’ Menus from the top 10 fast-food chains are nutritionally poor overall, they are of a slightly higher nutrition quality than those at cafés and restaurants. For example, most of the top 10 chain fast-food outlets offered fresh apple slices, carrot sticks, salad, yoghurt, low-fat milk or water as part of their Kids’ Menu [36]. Similarly, Serrano and colleagues found fast-food outlet Kids’ Menus (*n* = 10) had smaller portions and lower fat options compared with Kids’ Menus from non-fast-food outlets (*n* = 23) in a US community [37]. Clearly, additional supports are needed to improve the nutritional quality of Kids’ Menus in restaurants and cafés. 

Whilst only a handful of studies investigating Kids’ Meals within full service, sit-down restaurants have been undertaken internationally, our overall finding that Kids’ Menus are nutritionally very poor is similar to findings in other developed countries. For example, a US study assessed Kids’ Menus from 71 sit-down restaurants and found only 35% offered a healthy meal choice and less than half offered a healthy side option [38]. In Germany, an assessment of 1877 Kids’ Meals from 500 full-service restaurant menus found 70% of Kids’ Meals were one of six dishes of low nutritional quality [39]. Likewise in Northern Ireland, an assessment of 106 Kids’ Menus within restaurants found them to be limited in both menu choice and nutritional quality [40]. The poor nutritional quality of Kids’ Menu offerings in cafés and restaurants found in this study (and overseas) is concerning given Kids’ Menus typically target children 12 years and under, an age where life-long eating habits and taste preferences are formed [41]. Australian households eat out, on average, two-to-three nights per week [42], highlighting the need for restaurants and cafes to serve healthy, child-friendly meals to ensure that these experiences promote, rather than undermine, children’s health. 

The reasons why Kids’ Menus in restaurants and cafés are nutritionally very poor needs to be investigated further; however, recent interviews with restaurateurs offer some important insights. Anzman-Frasca and colleagues found that US restaurant owners perceived Kids’ Menus to be popular and accepted by children and their parents, but the healthier options were not a favorite among customers and only produced a small profit margin. [43] A key barrier to providing healthier Kids’ Meals was designing healthy meal options that appealed to children. Modifying menus was considered time-consuming and costly, and for that reason choices often remain unchanged [43]. Consumer demand and profit margin were also potential impetus for changing Kids’ Menus, as were government policies and corporate social responsibility [43]. 

Several cities in the US have mandated “health-by-default” ordinances, requiring restaurants to offer healthy beverages automatically with Kids’ Meals [44,45]. New York City also recently introduced a “Healthy Happy Meal Bill”, which sets nutrition standards for fast-food meals marketed to children with requirements to include fruits, vegetables, and whole grains and limit added sugar and salt [46]. Western Australian Local Governments are required to develop public health plans by 2023 that describe the actions they will take to support the health and wellbeing of the local community, working in partnership with population health experts from their area health service [47]. Creating and maintaining healthier food environments has been identified as an area to address, using tools such as the locally developed Food Outlet Dietary Risk assessment tool (FODR) to identify the risk of food outlets to public health nutrition [26]. Policy interventions to improve the nutritional quality of Kids’ Menus provides an opportunity for local governments to take action in this important area and report progress in the public health planning process. Working closely with restaurants and cafés through public–private partnerships, encouraging reformulation and providing rigorous voluntary nutrition standards for kids’ meals could improve impact [48]. It is promising to see in a study conducted by Alaya et al. [49] that the restaurant industry is open to working on reducing obesity in children and supporting public health efforts. This, coupled with Australian evidence that parents are ready to support implementation strategies to increase healthy options [50], demonstrates a ‘will’ for change.

Outside of multinational chain fast-food outlets, not much has been done to improve the nutritional quality of Kids’ Menus. Within the international literature, only a handful of community interventions exist. For example, the US National Kids LiveWell Program encourages chain restaurant Kids’ Menus to offer, as a minimum, one healthy main meal, one healthy side dish, healthy default-beverages and nutritional information available on request [51]. The Best Food for Families, Infants and Toddlers (Best Food FITS) intervention targets food businesses in Texas and involves dietitians reviewing existing Kids’ Menus and collaborating with restaurants to improve menus [52]. Although not specific to Kids’ Menus, Shape Up Somerville in the US worked with local restaurants to reduce portion sizes, add fruits and vegetables, and offer reduced-fat dairy products [53]; Steps to a Healthier Salinas targeted Taquerias to promote healthier foods [54]; and in San Antonio, ¡Por Vida! involved dietitians working with local restaurants to identify menu items that met Dietary Guidelines [55]. In 2015, South Australia Health formed a Healthy Kids Menu Taskforce designed to improve the quality and quantity of Kids’ Menus with a comprehensive mixed strategy approach. This consultation has now closed; however, a publicly available report provides an overview of possible strategies governments could support to improve menu quality for children [56]. Subsequently, South Australia Health, and Health and Wellbeing Queensland, launched a “Healthy Kids Menu” initiative which demonstrates that jurisdictions are prioritising cafes and restaurants in their mission to improve the quality of food environments for children [57]. Despite these efforts, rigorous studies identifying the most effective strategies for promoting the sales of healthy Kids’ Menu items are lacking and should be the focus of future research.

The strengths of this study are its large sample size (*n* = 787 restaurants and cafés, *n* = 258 Kids’ Menus audited in one geographic area), the use of two methodologies to assess the nutritional quality of Kids’ Menus (i.e., the Traffic Light System and the KIMEHS scoring tool). However, our sample was limited to a section of one Australian city, thus, the generalization of our results to other cities and countries is unknown. The nutritional quality assessment of Kids’ Menus was constrained by the limited information printed on the Kids’ Menus. Detailed descriptions of the ingredients and cooking methods were typically not provided, so assumptions had to be made. This may have led to inaccurate classifications of some food items (e.g., where Kids’ Menu listed ‘chicken nuggets and chips’ on the menu we assumed both the chicken nuggets and chips were deep fried; however, it is possible (but unlikely that these items were handmade and baked in the oven). Future studies may consider combining Kids’ Menu assessments with equipment audits and interviews with kitchen staff to determine the actual ingredients and cooking methods used for individual menu items. Assessment of portion size, total energy density, and the macro and micro-nutrient content of Kids’ Meals would also be useful, in addition to sales data, to determine the popularity of items. Consideration of other impacts of the food outlets was not included in this study as the focus was on nutritional quality of Kids’ Menus. However, future studies could incorporate the impact of food service outlets on local communities via increased traffic, litter, unpleasant smells, and antisocial behaviour [58].

## 5. Conclusions

This study appears to be the first in Australia to evaluate the nutritional quality of Kids’ Menus from restaurants and cafés. Overall, we found the nutritional quality of Kids’ Menus in both restaurants and cafés to be very poor. Concerningly, Kids’ Menus from cafés and restaurants were of poorer nutrition quality than Kids’ Menus from the top 10 fast-food chains (i.e., McDonalds, Hungry Jack’s, KFC, Domino’s, Red Rooster, Subway, Nando’s, Grill’d, Pizza Hut and Noodle Box). The findings demonstrate the need for additional supports to improve the nutritional quality of Kids’ Menus in restaurants and cafés. 

## Figures and Tables

**Table 1:** Proportion of Kids’ Menus in cafés and restaurants.

	Cafés (*n* = 382)		Restaurants (*n* = 405)		Cafés and Restaurants Combined (*n* = 787)	
	*N*	%	*N*	%	*N*	%
Food businesses with a separate Kids’ Menu	144	38	114	28	258	33
Food businesses with a separate Kids’ Menu AND children can order anything from the main menu at a reduced price and/or portion size	2	0.5	2	0.5	4	0.5
Food businesses without a separate Kids’ Menu	238	62	291	72	529	67
Food businesses with no separate Kids’ Menu but children could order anything from the main menu at a reduced price and/or portion size	62	16	46	12	108	14

**Table 2:** Number and price of main meals, side dishes, beverages, and desserts available on Kids’ Menus by food business type.

	Cafés (*n* = 144)		Restaurants (*n* = 114)		Cafés and Restaurants Combined (*n* = 258)	
	*n* (%)	Mean (SD), Range	*n* (%)	Mean (SD), Range	*n* (%)	Mean (SD), Range
Number of main meals present		5.0 (4.0), 0–16		4.2 (2.1), 1–13		4.6 (3.3), 0–16
Price of main meals (AUD)		9.10 (1.70) 3.00–16.00		12.16 (3.35) 6.00–28.00		10.15 (2.80) 3.00–28.00
Healthy default side option substitution allowed	2 (1%)		3 (3%)		5 (2%)	
Has a healthy default side (i.e., salad/vegetables)	3 (2%)		20 (18%)		23 (9%)	
Price increase for healthier default side	0 (0%)		1 (1%)		1 (0.4%)	
**Additional side dishes available**						
Kids’ Menus that offered additional side dishes	7 (5%)		15 (13%)		22 (9%)	
Total number of sides offered		0.6 (0.26), 0–2		0.18 (0.53), 0–3		0.11 (0.40), 0–3
Price of sides (AUD)		6.63 (1.98) 5.00–10.50		6.21 (1.46) 3.90–9.00		6.35 (1.62) 3.90–10.50
**Beverages**						
Total number of beverages offered		1.99 (1.97), 0–7		0.61 (1.04), 0–7		1.38 (1.76), 0–7
Water offered	1 (0.7%)		3 (3%)		4 (1.5%)	
Milk offered	18 (12%)		1 (1%)		19 (7%)	
Price of beverages (AUD)		3.70 (1.50), 1.00–6.30		4.20 (1.40), 1.00–7.50		3.70 (1.50), 1.00–7.50
**Desserts**						
Total number of desserts offered on Kids’ Menu		2.1 (2.4), 0–8		0.6 (0.9), 0–4		1.4 (2.1), 0–8
Price of desserts (AUD)		6.07 (2.32), 2.50–12.90		6.10 (2.82), 1.50–14.00		6.08 (2.37), 1.50–14.00
**Meal deals**						
Meal deal offered	48 (33%)		42 (37%)		90 (35%)	
**Marketing strategies**						
Recognizable logo or brand name present	37 (26%)		11 (10%)		48 (19%)	
Toy or ‘surprise’ offered	16 (11%)		6 (5%)		22 (9%)	
Cartoon character	0 (0%)		3 (3%)		3 (1%)	
Other marketing strategy (e.g., coloring in pack, use of pictures and colors)	33 (23%)		16 (14%)		49 (19%)	
**Nutritional information present**						
Total energy (kilojoules/kilocalories)	28 (19%)		5 (4%)		33 (13%)	
Other nutritional info (fat/sodium/fiber)	1 (0.7%)		3 (3%)		4 (2%)	
Allergen information	28 (19%)		24 (21%)		52 (20%)	
Healthy food items identified by symbol/words	2 (1.4%)		0		2 (1%)	
Healthier cooking methods identified on menu (e.g., grilled, steamed)	47 (33%)		12 (11%)		59 (23%)	

**Table 3:** Kids’ Menu items categorized by the Traffic Light System of food classification.

	Cafés (*n* = 144)			Restaurants (*n* = 114)			Cafés and Restaurants Combined (*n* = 258)		
Menu Component	% Green	% Amber	% Red	% Green	% Amber	% Red	% Green	% Amber	% Red
Main meals	23	0	77	31	2	67	26	1	73
Separate sides	13	0	88	10	0	91	10	0	90
Beverages	29	12	59	8	35	57	19	24	58
Desserts	19	16	65	6	0	94	17	13	70
All items combined	23	7	70	26	51	69	24	6	70

**Table 4:** Kids’ Menu Healthy Score (KIMEHS) results.

			KIMEHS Category									
	Total KIMEHS Score (Range −17 to 17)		Unhealthy (−17 to 0.49)		Moderately Unhealthy (0.5 to 5.49)		Going Healthy (5.5 to 11.49)		Moderately Healthy (11.5 to 13.49)		Healthy (13.5 to 17)	
	Mean (SD)	Range	*n*	%	*n*	%	*n*	%	*n*	%	*N*	%
Restaurants and Cafés combined (*n* = 258)	−8.5 (3.2)	−14.50, 3.50	256	99	2	1	0	0	0	0	0	0
All cafés (*n* = 144)	−9.5 (3.1)	−14.50, −1.25	144	100	0	0	0	0	0	0	0	0
All Restaurants (*n* = 114)	−7.2 (3.0)	−14.50, 3.50	112	98	2	2	0	0	0	0	0	0

**Table 5:** Nutrition assessment of the top 10 fast-food chain Kids’ Menu items using the Traffic Light System of food classification.

	Green	Amber	Red
	*n* (%)	*n* (%)	*n* (%)
Main meals	15 (34%)	3 (7%)	26 (59%)
Separate sides	6 (60%)	1 (10%)	3 (30%)
Beverages	6 (46%)	1 (8%)	6 (46%)
Desserts	0 (0%)	0 (0%)	0 (0%)
All Kids’ Menu items combined	28 (42%)	4 (6%)	35 (52%)

**Table 6:** Nutrition assessment of the top 10 fast-food chain Kids’ Menu items using the Kids’ Menu Healthy Score (KIMEHS).

Top 10 Fast-Food Chains	Total KIMEHS Score	Total KIMEHS Score Category
McDonald’s™	−1.5	Unhealthy
KFC™	−6.5	Unhealthy
Subway™	3	Moderately unhealthy
Hungry Jack’s™	−3	Unhealthy
Domino’s Pizza™	−6	Unhealthy
Red Rooster™	−5.5	Unhealthy
Grill’d™	−3	Unhealthy
Nando’s™	−4.5	Unhealthy
Pizza Hut™	−6	Unhealthy
Noodle Box™	−2	Unhealthy
	**Mean [SD], range**	
**All Top 10 fast-food chains combined**	−3.5 [2.89], −6.5 to 3	90% Unhealthy, 10% Moderately unhealthy

## Data Availability

Data supporting reported can be found by contacting the corresponding author.
